# *Planctoellipticum variicoloris* gen. nov., sp. nov., a novel member of the family *Planctomycetaceae* isolated from wastewater of the aeration lagoon of a sugar processing plant in Northern Germany

**DOI:** 10.1038/s41598-024-56373-y

**Published:** 2024-03-08

**Authors:** Carmen E. Wurzbacher, Tom Haufschild, Jonathan Hammer, Muriel C. F. van Teeseling, Nicolai Kallscheuer, Christian Jogler

**Affiliations:** 1 Department of Microbial Interactions, Institute of Microbiology, Friedrich Schiller University, Jena, Germany; 2 Junior Research Group “Prokaryotic Cell Biology”, Institute of Microbiology, Friedrich Schiller University, Jena, Germany

**Keywords:** Limnic bacteria, *Planctomycetota*, Asymmetric cell division, Schlesner strain collection, Pigmentation, Bacterial physiology, Bacterial genomics

## Abstract

In the present study, we characterise a strain isolated from the wastewater aeration lagoon of a sugar processing plant in Schleswig (Northern Germany) by Heinz Schlesner. As a pioneer in planctomycetal research, he isolated numerous strains belonging to the phylum *Planctomycetota* from aquatic habitats around the world. Phylogenetic analyses show that strain SH412^T^ belongs to the family *Planctomycetaceae* and shares with 91.6% the highest 16S rRNA gene sequence similarity with *Planctopirus limnophila* DSM 3776^T^. Its genome has a length of 7.3 Mb and a G + C content of 63.6%. Optimal growth of strain SH412^T^ occurs at pH 7.0–7.5 and 28 °C with its pigmentation depending on sunlight exposure. Strain SH412^T^ reproduces by polar asymmetric division (“budding”) and forms ovoid cells. The cell size determination was performed using a semi-automatic pipeline, which we first evaluated with the model species *P. limnophila* and then applied to strain SH412^T^. Furthermore, the data acquired during time-lapse analyses suggests a lifestyle switch from flagellated daughter cells to non-flagellated mother cells in the subsequent cycle. Based on our data, we suggest that strain SH412^T^ represents a novel species within a novel genus, for which we propose the name *Planctoellipticum variicoloris* gen. nov., sp. nov., with strain SH412^T^ (= CECT 30430^T^ = STH00996^T^, the STH number refers to the Jena Microbial Resource Collection JMRC) as the type strain of the new species.

## Introduction

Initially classified as eukaryotes^1^, members of the phylum *Planctomycetota* were only later acknowledged as bacteria^2^. Along with two eponymous and some other sister phyla, they form the *Planctomycetota-Chlamydiota-Verrucomicrobiota* (PVC) superphylum, whose ubiquitous members are of high medical and biotechnological relevance^3–5^. Similar to the other lineages of this superphylum, planctomycetes possess certain unique traits, some of which seemed to challenge the fundamental differences between pro- and eukaryotes and led to several alternative hypotheses about the evolution of eukaryotes^6^. For example, planctomycetes were proposed to lack peptidoglycan^7^, to form compartmentalised cells^8^, to possess a nucleus-like structure^9^, and to be capable of an endocytosis-like mechanism^10^, whereby especially the latter is a hallmark trait of eukaryotes. However, advances in high-resolution imaging techniques as well as the development of genetic tools for planctomycetes^11–13^ led to reinvestigations that induced a paradigm shift in planctomycetal research (for review see Wiegand et al.^6^). Thereby, planctomycetes were found to possess peptidoglycan^14,15^ and although exceptional in several other cell biological aspects, the cell envelope architecture of planctomycetes is considered like that of diderm (Gram-negative) bacteria^16–18^. Planctomycetal cells turned out to possess an enlarged periplasm with complex invagination patterns of the cytoplasmic membrane^16^. Combined with the crateriform structures found on the surface of various planctomycetes, these invaginations are presumably involved in a yet uncharacterised mechanism for the uptake of entire high molecular weight polysaccharide molecules and their degradation^6,16^.

Although some of the controversy is now resolved, members of the phylum *Planctomycetota* are still exceptional. They employ different division modes, either a mode of asymmetric polar reproduction (“budding”) (class *Planctomycetia*), binary fission (classes *Phycispherae* and *Candidatus* Brocadiae), or a combination of both types^19^, while lacking most of the canonical bacterial cell division enzymes including the contractile Z-ring-forming key player FtsZ^19–21^. Some strains, as for example the model species *Planctopirus limnophila*, have been reported to undergo a lifestyle switch, with attached cells reproducing asymmetrically at the pole opposite to the surface-anchored holdfast structure. Newly produced daughter cells are flagellated and swim until they attach to a surface and start to divide themselves^6,11,22,23^.

Besides their uncommon cell biology, planctomycetal genomes are just as intriguing and hold potential for biotechnological application of this phylum. In comparison to members of other bacterial phyla, planctomycetes can possess exceptionally large genomes of up to 12.4 Mb (*Fimbriiglobus ruber*) and are the phylum containing the most predicted genes with unknown function^6,24,25^. Genome sizes also differ highly between the currently described classes of the phylum (*Planctomycetia*: 4.8–12.4 Mb, *Phycisphaerae* and *Ca*. Brocadiae: 3.0–4.3 Mb). As shown in the past years, members of the phylum *Planctomycetota* produce small molecules with possible health-promoting bioactivities^5,26–29^. Among the small molecules recently discovered in planctomycetes are stieleriacines^30–32^, carotenoids^33,34^ and an aromatic compound potentially acting as a plant toxin^35^.

Here, we characterise strain SH412^T^, which was isolated by Heinz Schlesner (Institute for Microbiology, Christian-Albrechts-University, Kiel, Germany) in the late 1980s from the wastewater aeration lagoon of a sugar processing plant in Schleswig, a town in Northern Germany. Strain SH412^T^ is one of approximately 257 strains whose isolation has been reported in 1994^36^. However, a polyphasic analysis of phenotypic and genomic features has not yet been conducted for many strains from Schlesner’s collection^37^. In addition, we present a standardised, semi-automatic pipeline for cell size determination employing light microscopy, which was first tested with the limnic model planctomycete *Planctopirus limnophila* DSM 3776^T^ and then applied to the novel strain.

## Materials and methods

### Isolation and cultivation

Strain SH412^T^ was isolated by Heinz Schlesner (Institute for Microbiology, Christian-Albrechts-University, Kiel, Germany) from a wastewater aeration lagoon of a sugar processing plant in Schleswig (Northern Germany). At the sampling site, a conductivity of 6270 µS/cm and a pH of 7.8 were measured. The strain was isolated on M31PY medium and routinely cultivated at 28 °C in the same medium. *P. limnophila* DSM 3776^T^ served for testing of the cell size determination pipeline and was cultivated in limnic M1 medium at 28 °C. 1 L M31PY medium contained Mineral Salt Solution (Hutner’s Basal Salts, 20 mL, see below), BD Bacto Peptone (0.25 g), BD Bacto Yeast Extract (0.25 g), Tris/HCl (final concentration 5 mM), CaCl_2_ × 2 H_2_O (0.1 g) and MgCl_2_ × 6 H_2_O (0.1 g). The pH was adjusted to 7.5 in liquid cultures and pH 8.5 for agar plates. After autoclaving, the following amounts of sterile-filtered solutions were added: *N*-acetyl glucosamine solution (40 mL of 50 g/L stock), vitamin solution (5 mL, see below) and Na_2_HPO_4_ × 2 H_2_O solution (1 mL of 100 g/L stock). For solidified medium, 15 g/L agar, previously washed three times with ddH_2_O and separately autoclaved, were added. 1 L of limnic M1 medium contained Mineral Salt Solution (Hutner’s Basal Salts, 20 mL, see below), NH_4_Cl (0.53 mg), KH_2_PO_4_ (1.4 mg), KNO_3_ (10 mg), MgSO_4_ × 7 H_2_O (49.3 mg), CaCl_2_ × 2 H_2_O (14.7 mg), CaCO_3_ (25 mg), NaHCO_3_ (25 mg), BD Bacto Peptone (0.25 g), BD Bacto Yeast Extract (0.25 g), and 4-(2-hydroxyethyl)-1-piperazineethanesulfonic acid (HEPES, 2.38 g). The pH value was adjusted to 7.0. After autoclaving, the following sterile-filtered solutions were added: glucose solution (4 mL, 25% (v/w) stock), vitamin solution (5 mL, see below), *N*-acetyl glucosamine (20 mL, 50 g/L stock), and trace element solution (1 mL, see below). The following supplemental solutions were prepared for both media: 1 L vitamin solution contained *p*-aminobenzoic acid (0.01 g), biotin (0.004 g), pyridoxine hydrochloride (0.02 g), thiamine hydrochloride (0.01 g), sodium pantothenate (0.01 g), folic acid (0.004 g), riboflavin (0.01 g), nicotinamide (0.01 g) and vitamin B_12_ (0.0002 g). 1 L Mineral Salt Solution (Hutner’s Basal Salts) contained nitrilotriacetic acid (10 g), MgSO_4_ × 7 H_2_O (29.7 g), CaCl_2_ × 2 H_2_O (3.34 g), FeSO_4_ × 7 H_2_O (0.099 g) and Metal Salt Solution 44 (50 mL). 1 L of Metal Salt Solution 44 contained Na_2_-EDTA (250 g), ZnSO_4_ × 7 H_2_O (1.095 g), FeSO_4_ × 7 H_2_O (0.5 g), MnSO_4_ × H_2_O (0.154 g), CuSO_4_ × 5 H_2_O (0.0395 g), CoCl_2_ × 6 H_2_O (0.0203 g) and Na_2_B_4_O_7_ × 10 H_2_O (0.0177 g). 1 L Trace Element Solution (used only for limnic M1 medium) contained sodium nitrilotriacetate (1.5 g), MnSO_4_ × 7 H_2_O (0.5 g), FeSO_4_ × 7 H_2_O (0.1 g), Co(NO_3_)_2_ × 6 H_2_O (0.1 g), ZnCl_2_ (0.1 g), NiCl_2_ × 6 H_2_O (0.05 g), H_2_SeO_3_ (0.05 g), CuSO_4_ × 5 H_2_O (0.01 g), AlK(SO_4_)_2_ × 12 H_2_O (0.01 g), H_3_BO_3_ (0.01 g), NaMoO_4_ × 2 H_2_O (0.01 g), Na_2_WO_4_ × 2 H_2_O (0.01 g).

### Initial PCR-based amplification of the 16S rRNA gene

An amplification and sequencing of the 16S rRNA gene of the novel isolate was performed to determine its phylogenetic position and degree of novelty. The sequencing was also used to check the purity of the axenic culture. Colonies from plates were lysed by boiling (95 °C, 3 min) and subsequent freezing (three times repeated). Prior to genome sequencing, extracted gDNA was used as PCR template to check for purity. The following primers were used for the amplification reaction: eubplancto8f 5′-AGAGTTTGATCMTGGCTCAG-3′ and eubplancto1492r 5′-GGYTACCTTGTTACGACTT-3′. Dream Taq DNA Polymerase (Thermo Scientific) was employed for the reaction and the PCR was performed using the following program: Initial denaturation for 2 min at 95 °C, followed by 45 cycles of denaturation (30 s at 95 °C), annealing (30 s), and elongation (2 min at 72 °C). Thereby the annealing temperature was 54 °C during the first ten and 49 °C during the remaining 35 cycles. Afterwards, the PCR was concluded by a final elongation step for 7 min at 72 °C. For sequencing, samples were prepared with the primer eubplancto8f to obtain a partial 16S rRNA sequence. Sequencing was performed by Macrogen Europe.

### Isolation of genomic DNA

Genomic DNA for long-read sequencing was isolated using the Wizard HMW DNA Extraction Kit (Promega) following the manufacturer’s instructions. The obtained gDNA was quantified using the Qubit fluorometer with the Qubit dsDNA BR-Assay-Kit (Thermo Scientific). DNA quality and purity were determined using NanoDrop 2000 (Thermo Scientific) absorbance measurements at 230, 260 and 280 nm. Integrity and fragment length distribution of obtained gDNA were analysed with the 4150 TapeStation System using Genomic DNA reagents and ScreenTape (Agilent Technologies).

### Long-read sequencing with Oxford Nanopore and genome assembly

For nanopore long-read sequencing, a multiplex sequencing library was prepared from at least 400 ng of high quality gDNA per sample according to the manufacturer’s protocol for the Native Barcoding Kit 24 (SQK-NBD112.24, Oxford Nanopore Technologies). Briefly, the DNA was repaired using the NEBNext FFPE DNA Repair Mix and the NEBNext Ultra II End Repair/dA-tailing Module reagents (New England Biolabs). A clean-up was performed with one volume of AMPure XP Beads (Beckman Coulter) per sample volume. The equimolar mass of each DNA sample was taken forward to the native barcode ligation step using the NEB Blunt/TA Ligase Mix (New England Biolabs) and the ligation was stopped using EDTA. Barcoded DNA of all samples was pooled and cleaned with 0.4 volumes of AMPure XP Beads (Beckman Coulter). Sequencing adapters were ligated to the DNA using the NEBNext Quick Ligation Module (New England Biolabs) and another purification step with 0.4 volumes of beads was performed. The washing steps were performed with Long Fragment Buffer (Oxford Nanopore Technologies) instead of 70% (v/v) ethanol to enrich DNA fragments larger than 3 kb. For sequencing, 5 to 10 fmol of the final library were loaded onto a primed R10.4 flowcell (FLO-MIN112, Oxford Nanopore Technologies). Sequencing was performed on a MinION Mk1B device (Oxford Nanopore Technologies). The raw nanopore sequencing data was basecalled using bonito version 0.5.3 with the basecalling model dna_10.4_e8.1_sup@v3.4 (Oxford Nanopore Technologies). The basecalled reads were filtered using NanoFilt 2.8.0^38^ and only reads with a Phred quality score ≥ 10 were further processed. Demultiplexing and adapter trimming were performed using Guppy version 6.3.9 with the optional flag "–trim_barcodes" (Oxford Nanopore Technologies). Demultiplexed and trimmed reads were uploaded to the Galaxy web platform and the server available under the public domain usegalaxy.eu was used for the processing of the data^39^. We created and used a Galaxy workflow that is available at https://usegalaxy.eu/u/domi/w/copy-of-fastqflyemedakaprokkabuscoquast. The workflow included NanoPlot version 1.28.2 for quality control of the nanopore reads^38^, Flye version 2.9^40,41^ for genome assembly and Medaka version 1.4.4 (Oxford Nanopore Technologies) for polishing of the raw assembly with the original long reads. The workflow also includes evaluation of the assembly completeness using BUSCO version 5.2.2^42,43^ and annotation of the assembled genome using Prokka version 1.14.6^44,45^.

### Polishing of the assembly with Illumina short reads and post-processing

Library preparation and short-read sequencing using the Illumina NovaSeq platform was performed by Eurofins Genomics Germany GmbH. The Illumina reads (approx. 5 million read pairs, 2 × 150 bp) per strain were also uploaded to the Galaxy web server usegalaxy.eu^39^. We created and used another Galaxy workflow that is available via https://usegalaxy.eu/u/domi/w/copy-of-bwa-mem2pilonprokkabuscoquast. The workflow comprises evaluation of the Illumina reads using FastQC version 0.11.9 (https://www.bioinformatics.babraham.ac.uk/projects/fastqc/), mapping of Illumina reads to the genome assembly using BWA-MEM2 version 2.2.1^46–48^ and polishing using Pilon version 1.20^49^. Evaluation of assembly completeness was performed again with BUSCO version 5.2.2^42,43^. The polished assembly was again annotated using Prokka version 1.14.6^44,45^. The obtained genome was manually circularised and rotated to the start codon of the chromosomal replication initiator protein-encoding gene *dnaA* (according to the Prokka annotation). The genome was finally annotated using the NCBI Prokaryotic Genome Annotation Pipeline (PGAP) version 2022–12-13.build6494^50–52^.

### Nucleotide sequence accession numbers

The 16S rRNA gene and genome sequence of strain SH412^T^ are available from the NCBI GenBank database under the accession numbers OR353748 and CP130886, respectively.

### Phylogenomic analyses

The 16S rRNA gene sequence of strain SH412^T^ was extracted from the annotated genome and the identification of the closest neighbours of the novel isolate was performed using NCBI’s BLASTn suite (accessed on 5th July 2023)^53^. The 16S rRNA gene sequences of strain SH412^T^ and all characterised members of the phylum were aligned with ClustalW^54^. The alignment was used to calculate a 16S rRNA similarity matrix with TaxonDC^55^. The 16S rRNA gene sequence-based maximum likelihood phylogenetic tree was calculated from the same alignment with FastTree 2.1^56^ employing the GTR + CAT model and 1000 bootstraps replications. Three 16S rRNA genes of bacterial strains from the PVC superphylum outside of the phylum *Planctomycetota*, namely *Opitutus terrae* (NCBI acc. no. AJ229235), *Kiritimatiella glycovorans* (acc. no. NR_146840) and *Lentisphaera araneosa* (acc. no. NR_027571), were used as outgroup. The multi-locus sequence-based phylogenetic analysis was performed using autoMLST with 500 bootstrap replicates^57^. The analysis was performed with the autoMLST-simplified-wrapper tool available on GitHub. The MLSA-based phylogenetic tree was constructed based on the reference genomes of all current members of the family *Planctomycetaceae* (GenBank accession numbers are given in brackets): *Alienimonas californiensis* CA12^T^ (GCA_007743815.1), *Alienimonas chondri* LzC2^T^ (GCA_013036045.1), *Calycomorphotria hydatis* V22^T^ (GCA_007745435.1), *Caulifigura coniformis* Pan44^T^ (GCA_007745175.1), *Fuerstiella marisgermanici* NH11^T^ (GCA_001983935.1), *Gimesia alba* Pan241w^T^ (GCA_007744675.1), *Gimesia algae* Pan161^T^ (GCA_007746795.1), *Gimesia aquarii* V144^T^ (GCA_007748195.1), *Gimesia benthica* E7^T^ (GCA_009720525.1), *Gimesia chilikensis* JC646^T^ (GCA_008329715.1), *Gimesia fumaroli* Enr17^T^ (GCA_007754425.1), *Gimesia maris* CA11 (GCA_007747015.1), *Gimesia panareensis* Pan110^T^ (GCA_007748015.1), *Maioricimonas rarisocia* Mal4^T^ (GCA_007747795.1), *Planctomicrobium piriforme* P3^T^ (GCA_900113665.1), *Planctopirus ephydatiae* spb1^T^ (GCA_007752345.1), *Planctopirus hydrillae* JC280^T^ (GCA_001707835.1), *Planctopirus limnophila* DSM 3776^T^ (GCA_000092105.1), *Rubinisphaera brasiliensis* DSM 5305^T^ (GCA_000165715.3), *Rubinisphaera italica* Pan54^T^ (GCA_007859715.1), *Rubinisphaera margarita* ICM_H10^T^ (GCA_022267515.1), *Schlesneria paludicola* MPL7^T^ (GCA_000255655.1), *Symmachiella dynata* Mal52^T^ (GCA_007747995.1), *Symmachiella dynata* Pan258 (GCA_007744975.1), *Symmachiella macrocystis* CA54^T^ (GCA_007860075.1), *Thalassoglobus neptunius* KOR42^T^ (GCA_007859735.1), *Thalassoglobus polymorphus* Mal48^T^ (GCA_007744255.1) and *Thalassoroseus pseudoceratinae* JC658^T^ (GCA_011634775.1). The genomes of *Rhodopirellula baltica* SH1^T^ (GenBank acc. no. BX119912.1), *Pirellula staleyi* DSM 6068^T^ (acc. no. CP001848.1) and *Blastopirellula marina* DSM 3645^T^ (acc. no. GCA_000153105.1) (all belonging to the family *Pirellulaceae*) served as outgroup. Average amino acid identities (gAAI) and average nucleotide identities (gANI) were calculated using the respective scripts of the enveomics collection^58^. The percentage of conserved proteins (POCP) was calculated as described^59^. The *rpoB* gene sequences were taken from publicly available genome annotations and sequence identities were determined as previously described^60^. The alignment and matrix calculation were performed upon extracting a 1298 bp region of the *rpoB* coding sequence that would have been sequenced with the described primer set. Alignment and matrix calculation were performed with Clustal Omega^61^.

### Analysis of genome-encoded features

The “Estimate Metabolism” function of anvi’o v. 7.1^62^ was used for the analysis of genome-encoded primary metabolic functions. Numbers of putative carbohydrate-active enzymes (CAZymes) were obtained from the genome annotation provided by eggnog-mapper v.2.1.10^63^. An in silico prediction of biosynthetic gene clusters (BGCs) putatively involved in the biosynthesis of secondary metabolites was carried out using antiSMASH 7^64^. The prediction was run with relaxed strictness, antiSMASH beta features and all extra features activated. The genome completeness was assessed with BUSCO v5.4.7^43^, while the coding density was analysed with CheckM v1.1.6^65^.

### Physiological analysis

For determination of the temperature optimum for growth, 100 µL supernatant of an exponentially growing culture (containing no visible aggregates) were plated and plates were incubated in triplicates at temperatures ranging from 4 to 37 °C. Plates were checked daily, and growth was evaluated by the time required until visible colonies were formed. The temperature at which colonies appeared the earliest was considered the temperature optimum for growth. The pH optimum for growth was determined in liquid cultures. For this purpose, Tris-buffer in M31PY was substituted by 100 mM of buffering agents 2-(*N*-morpholino)ethanesulfonic acid (MES) for pH 5.0 and 6.0, 4-(2-hydroxyethyl)-1-piperazineethanesulfonic acid (HEPES) for pH 7.0 and 8.0 and *N*-cyclohexyl-2-aminoethanesulfonic acid (CHES) for pH 9.0 and 10.0, whereas for pH 7.5 standard M31PY liquid medium was used. Supernatant from a growing culture was used for inoculation and cultivation was performed at 24 °C in duplicates without agitation. The temperature was chosen after determining the temperature optimum for growth of strain SH412^T^ and displays a compromise between fastest growth and most biomass production (see below). Growth was evaluated by eye by the amount of aggregates (biomass) formed after 18 days incubation, as optical density (OD) measurements are not possible with this strain. Anaerobic growth was examined on a plate that was incubated at 21 °C in an anaerobic jar. The presence of catalase was tested by transferring a colony of the strain into a drop of 3% (v/v) H_2_O_2_; the test is considered positive upon vigorous gas production. The presence of cytochrome *c* oxidase was tested with Kovács oxidase test reagent on filter paper, where a colour change towards dark blue indicates a positive test result.

### Light microscopy

Phase-contrast and differential interference contrast (DIC) analyses were performed with a Nikon Eclipse Ti2 inverted microscope, with a Nikon DS-Ri2 Camera and a Nikon CFI Plan Apochromat Lambda 100X oil immersion objective (numerical aperture 1.45). For phase contrast analyses the objective additionally contained a Ph3 module. The Images were processed employing the Nikon NIS-Elements software (Version 5.30, https://www.microscope.healthcare.nikon.com/de_EU/products/software/nis-elements) and FIJI (Version 2.9.0, https://downloads.imagej.net/fiji/releases/2.9.0/). Specimens were immobilised on a medium-supplemented 1% (w/v) agarose cushion, for time-lapse analyses the cover glass was sealed with VLAP (33% (w/w) vaseline, 33% (w/w) lanoline, 33% (w/w) paraffin) against the slide to minimise evaporation. Additional time-lapse analyses were performed in a Nunc Glass Base Dish (Thermo Fisher Scientific, 12 mm), which was incubated with supernatant of a growing culture for 24 h. Fluid was then discarded, and remaining cells were immobilised with an agarose cushion as described above.

### Cell size determination

To test the new cell size determination pipeline, a pre-culture of the model strain *P. limnophila* DSM 3776^ T^ was grown to the mid-exponential phase and then split into independent replicates. For microscopy, these replicates were then grown until early exponential phase. This procedure ensured a reproducible cell size and prevented an overcrowded image during microscopy. Phase-contrast images were taken as described above. Since each image contained three channels (RGB), for further image analysis the second channel was duplicated and saved as a separate tiff file in FIJI. Length and width of the exact same 100 cells were analysed employing three different methods: Firstly, two independent researchers measured the cells using the line and measurement tool in FIJI^66^. Then, MicrobeJ (Version 5.13 m)^67^ and BacStalk (Version 1.8)^68^ were employed for size determination. For cell detection in MicrobeJ, the thresholding was set to bright, auto, and default mode. The area was set to 1-max, length to 0.9–3, width to 0.5–2.5, and all other parameters were set to 0-max. Length and width of the detected cells were determined in medial axis mode. To analyse the cells in BacStalk, the respective images of one replicate were loaded into the software. The pixel size was set to 0.029 µm, the option for stalk detection was unchecked. The segmentation parameters were set to 20 pixels for cell size and 15 pixels for minimum cell size. All budding cells and aggregates were manually removed from the analysis and only solitary cells were analysed. The obtained data was transformed from .csv files to .xlsx files and uploaded separately into SuperPlotsOfData. For strain SH412^T^ the cell size determination was done for three independently growing cultures (incubated at 28 °C) and 150 cells from each of the supernatants were analysed as described above.

### Analysis of light-dependent pigmentation

To investigate an influence of light on the degree of pigmentation of the strain, duplicates were inoculated on agar plates and incubated in the same room either directly next to a window or covered in aluminium foil inside a cabinet.

## Results and discussion

### Phylogenetic analysis

**Figure 1:**
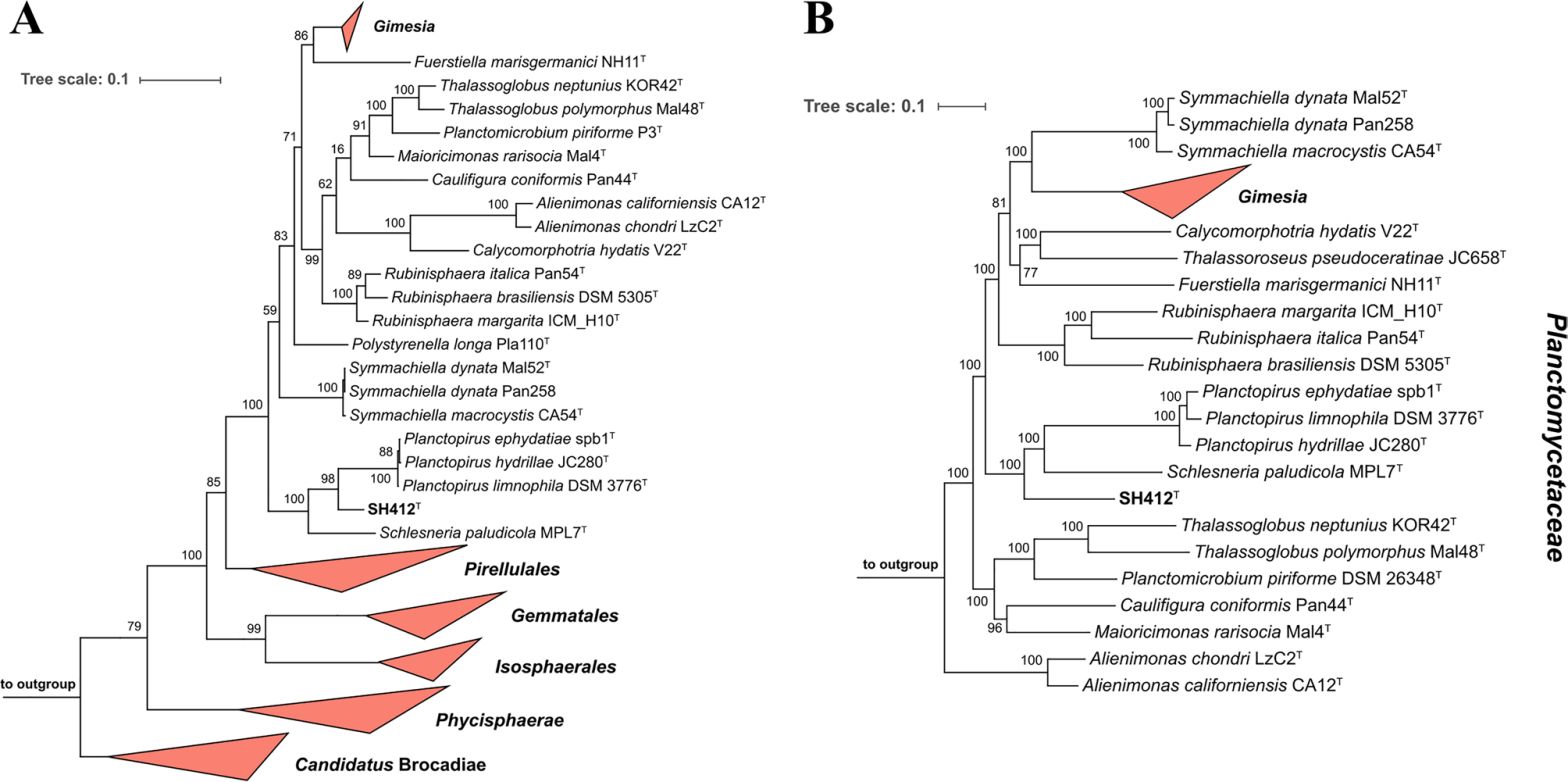
Maximum likelihood 16S rRNA gene sequence- (**A**) and MLSA-based (**B**) phylogenetic trees highlighting the position of SH412^T^. Analyses were performed as described in the Materials and Methods section. Bootstrap values after 1000 (16S rRNA gene sequence-based tree) or 500 re-samplings (MLSA-based tree) are given at the nodes (in %).

The phylogenetic position of strain SH412^T^ was determined based on its 16S rRNA gene sequence and MLSA (Fig. 1). In both constructed maximum likelihood phylogenetic trees, strain SH412^T^ clusters in the family *Planctomycetaceae* close to the characterised members of the genera *Planctopirus* and *Schlesneria*. While in the 16S rRNA gene sequence-based tree SH412^T^ clusters between the two genera, in the MLSA-based tree it clusters outside of them. Both tree positions display good bootstrap support (96–100%). To further analyse the phylogenetic position of SH412^T^, five phylogenetic markers were evaluated: 16S rRNA gene sequence similarity, gANI, gAAI, POCP and similarity of a 1298 bp partial sequence of the gene *rpoB* coding for the β-subunit of the RNA polymerase, which is an established phylogenetic marker within the order *Planctomycetales*^60^. The maximal gANI value of 76.7% between SH412^T^ and the four closest neighbours showed that the strain does not belong to a described species (threshold of 95%^69^). Strain SH412^T^ shares the highest 16S rRNA gene sequence similarity with *P. limnophila* DSM 3776^T^ (91.6%). Thus, taking the 16S rRNA gene similarity genus threshold of 94.5%^70^ into account, SH412^T^ belongs to a novel genus.

In contrast, comparison of the SH412^T^ partial *rpoB* gene with members of the genus *Planctopirus* yielded values between 75.9 and 76.2%. Such values are in the genus threshold range of 75.5–78.0%^71^. However, the value obtained for a comparison with *S. paludicola* MPL7^T^ (79.1%) is slightly above the genus threshold. Yet, results from the other phylogenetic analyses support SH412^T^ as a new species of a distinct genus: a maximal gAAI value of 56.9% is obtained with *S. paludicola* MPL7^T^; with members of the genus *Planctopirus* values around 54% are obtained. These values are far below the genus threshold range of gAAI (60–80%)^72,73^. The same is true for the obtained POCP values (< 48.7%) which are below the accepted genus threshold of 50%^59^. Although the analysed phylogenetic markers imply different species as the current next relative of strain SH412^T^, four out of five markers suggest that the strain should be delineated from the described genera in the family *Planctomycetaceae*, forming a novel genus and species.

### Genomic characteristics

The type strains of the two closest related genera were chosen for the comparison of genomic features of the novel isolate: *P. limnophila* DSM 3776^T^^74^, *P. ephydatiae* spb1^T^^75^, *P. hydrillae* JC280^T^^76^, and *S. paludicola* MPL7^T^^77^ (Table 1). The genome of strain SH412^T^ is 7,276,092 bp in size and has a DNA G + C content of 63.6%. It is thereby considerably larger than the genomes of the current genus *Planctopirus* (5.2–5.8 Mb) but smaller than the one of *S. paludicola* MPL7^T^ (8.7 Mb). Extrachromosomal elements are not present. Notably, the DNA G + C content of strain SH412^T^ is almost 10 percentage points higher than in its currently closest relatives. Relevant additional genomic features are summarised in Table 1. The automatic gene annotation by NCBI’s Prokaryotic Genome Annotation Pipeline (PGAP) predicted 5608 protein-coding genes, of which 28% (1573 genes) are categorised as hypothetical proteins. In accordance with its larger genome, the number of predicted protein-coding genes of strain SH412^T^ lies between the values of the genera *Planctopirus* and *Schlesneria*. However, strain SH412^T^ has both the highest number of genes per Mb as well as highest coding density, although all values are within the same range. Furthermore, strain SH412^T^ carries two copies of each rRNA gene, while most of its closest relatives merely possess multiple copies of one of these genes. Remarkably, the genome of strain SH412^T^ contains only 54 tRNA genes, while all the other strains possess at least 20 more tRNA genes.

**Table 1:** Comparison of genomic characteristics of strain SH412^T^ and the type strains of *Planctopirus limnophila*, *Planctopirus ephydatiae*, *Planctopirus hydrillae*, and *Schlesneria paludicola* regarding genomic features.

Characteristics	SH412^T^	*Planctopirus limnophila* DSM 3776^T^	*Planctopirus ephydatiae* spb1^T^	*Planctopirus hydrillae* JC280^T^	*Schlesneria paludicola* MPL7^T^
Genome size [bp]	7,276,092	5,460,085	5,214,061	5,750,243	8,702,386
Plasmids	0	1	0	n.d.	n.d.
DNA G + C [%]	63.6	53.5	53.8	53.8	55.7
Coding density [%]	85.9	84.9	85.2	84.5	83.9
Completeness (Busco) [%]	99.8	99.5	99.3	98.2	99.7
Genes (total)	5693	4109	3909	4357	6306
Genes/Mb	782	753	750	758	725
Protein-coding genes	5608	4015	3818	4248	6181
Protein-coding genes/Mb	771	735	732	739	710
Hypothetical proteins	1573	1071	945	1230	1640
Hypothetical proteins [%]	28.0	26.7	24.8	29.0	26.5
rRNAs (5S, 16S, 23S)	2, 2, 2	1, 2, 1	1, 2, 1	1, 1, 1	3, 1, 1
tRNAs	54	74	75	75	77
Carbohydrate-active enzymes					
Glycoside hydrolases	17	11	11	11	19
Glycosyl transferases	22	18	17	21	23
Carbohydrate esterases	2	1	6	1	4
Carbohydrate-bind. Modules	8	5	5	5	4
antiSMASH Biosynthetic gene clusters					
Terpenoid	3	3	3	3	3
Type I PKS	2	3	4	3	4
Type III PKS	0	1	1	1	2
NRPS-like	0	0	1	0	2
Resorcinol	0	0	1	0	0
Arylpolyene	0	0	1	0	0
Bacteriocin	0	1	0	0	1
RiPP-like	2	1	0	0	1

*n.d*. not detected.

### Analysis of genome-encoded features

In order to predict metabolic capabilities of strain SH412^T^, the genome-based primary metabolism of the novel isolate was analysed with the “Estimate Metabolism” tool of anvi’o. All analyses in the following section were also performed again for the four type strains of the currently closest related genera (*P. limnophila* DSM 3776^ T^, *P. ephydatiae* spb1^T^, *P. hydrillae* JC280^T^, and *S. paludicola* MPL7^T^), as this information was, given the historical context, either not available at all for some strains or different software/older software versions had been used previously. The results point towards a canonical central metabolism expected in a heterotrophic bacterium. A complete set of enzymes required for a functional glycolysis, tricarboxylic acid (TCA) cycle, pentose phosphate pathway and respiratory chain is encoded in the genome. The strain harbours a gene coding for cytochrome *c* oxidase and but lacks *catA* (also referred to as *katA)* encoding catalase. A putative catalase superfamily protein was annotated by PGAP, but the protein lacks several domains that are typically found in catalases. These results from the genome annotation are in line with the results obtained for the respective enzyme activity tests (Table 2). Based on the functional annotation the strain should be capable of the de novo biosynthesis of fatty acids, nucleotides, proteinogenic amino acids and isoprenoids. At least two genes of strain SH412^T^ code for vitamin B_12_ (cobalamin)-dependent enzymes, namely the methionine synthase MetH and the class II ribonucleotide reductase NrdJ. Since the genome indicates the lack of a functional pathway for biosynthesis of this vitamin, the strain is likely auxotrophic for vitamin B_12_. The same was shown for the close relative *P. limnophila*^78^, but no data is available for the other three strains chosen for comparison.

**Table 2:** Comparison of phenotypic characteristics of strain SH412^T^ and the type strains of *Planctopirus limnophila*, *Planctopirus ephydatiae*, *Planctopirus hydrillae*, and *Schlesneria paludicola*.

Characteristics	SH412^T^	*Planctopirus limnophila* DSM 3776^T^	*Planctopirus ephydatiae* spb1^T^	*Planctopirus hydrillae* JC280^T^	*Schlesneria paludicola* MPL7^T^
Literature	This study	^11^, ^74^, ^76^	^75^	^76^	^77^
Colour	Ranging from almost white to dark pink/red, varying orange, red, and pink components	Red	Pink	Dark pink	Unpigmented
Size (µm)	0.7–2.2 × 0.7–2.8	1.1–1.5	1.1–1.5 × 1.5–2.5	1.0–1.4	0.8–1.4 × 1.3–2.1
Shape	Primarily ovoid, small portion almost spherical	Spherical to ovoid	Ovoid to spherical	Spherical to ovoid	Ellipsoid
Clusters	Large aggregates, “flakes”, strong cohesion	Aggregates	Rosettes, aggregates	Aggregates	Pairs, rosettes
Temperature range (optimum) (C°)	10–32 (28)	17–39 (30–32)	15–33 (30)	10–45 (20–30)	4–32 (20–28/15–26)*
pH range (optimum)	6.0–8.0 (7.0–7.5)	6.2–8.0 (6.2–7.0)	7.0–9.0 (9.0)	6.0–9.0 (6.5–7.5)	4.2–7.2 (5.0–6.2)
Division	Asymmetric	Asymmetric	Asymmetric	Asymmetric	Asymmetric
Dimorphic lifestyle	Yes**	Yes	Yes	n.i.	Yes
Motility/flagella	Yes/genes present	Yes/monotrichous, polar	Yes/n.i	Yes/monotrichous, sub-polar	Yes/lophotrichous, two flagella
Respiration	Facultative anaerobic	Facultative anaerobic	n.i.	Obligate aerobic	Facultative anaerobic
Catalase	Negative	n.i.	n.i.	Positive	Positive
Cytochrome *c* oxidase	Positive	n.i.	n.i.	Positive	Positive

*n.i.* no information available.

*Discrepancies between values in paper and supplementary information, **not verified.

Besides the central metabolism, we also analysed the genomes for putative carbohydrate-active enzymes (CAZymes) and biosynthetic gene clusters (BGCs), since members of the phylum *Planctomycetota* are expected to be of high biotechnological relevance^6^. The numbers of CAZymes of the analysed strains fall between 35 and 50. The obtained numbers turned out to reflect the size of the genomes. Members of the genus *Planctopirus* have less than 40 putative CAZymes, whereas strain SH412^T^ and *S. paludicola* MPL7^T^, both with considerable larger genomes, have 49 or 50 candidate CAZymes. Glycoside hydrolases and glycosyltransferases are the dominant classes of CAZymes in all four species.

Genome mining for BGCs by antiSMASH provides indications that strain SH412^T^ produces terpenoids, polyketide compounds derived from type I polyketide synthases and ribosomally synthesised and post-translationally modified peptides. Despite the larger genome of strain SH412^T^ compared to the type strains of the three *Planctopirus* species, it has an equal or even lower number of BGCs.

### Macroscopic appearance and physiological characteristics

**Figure 2:**
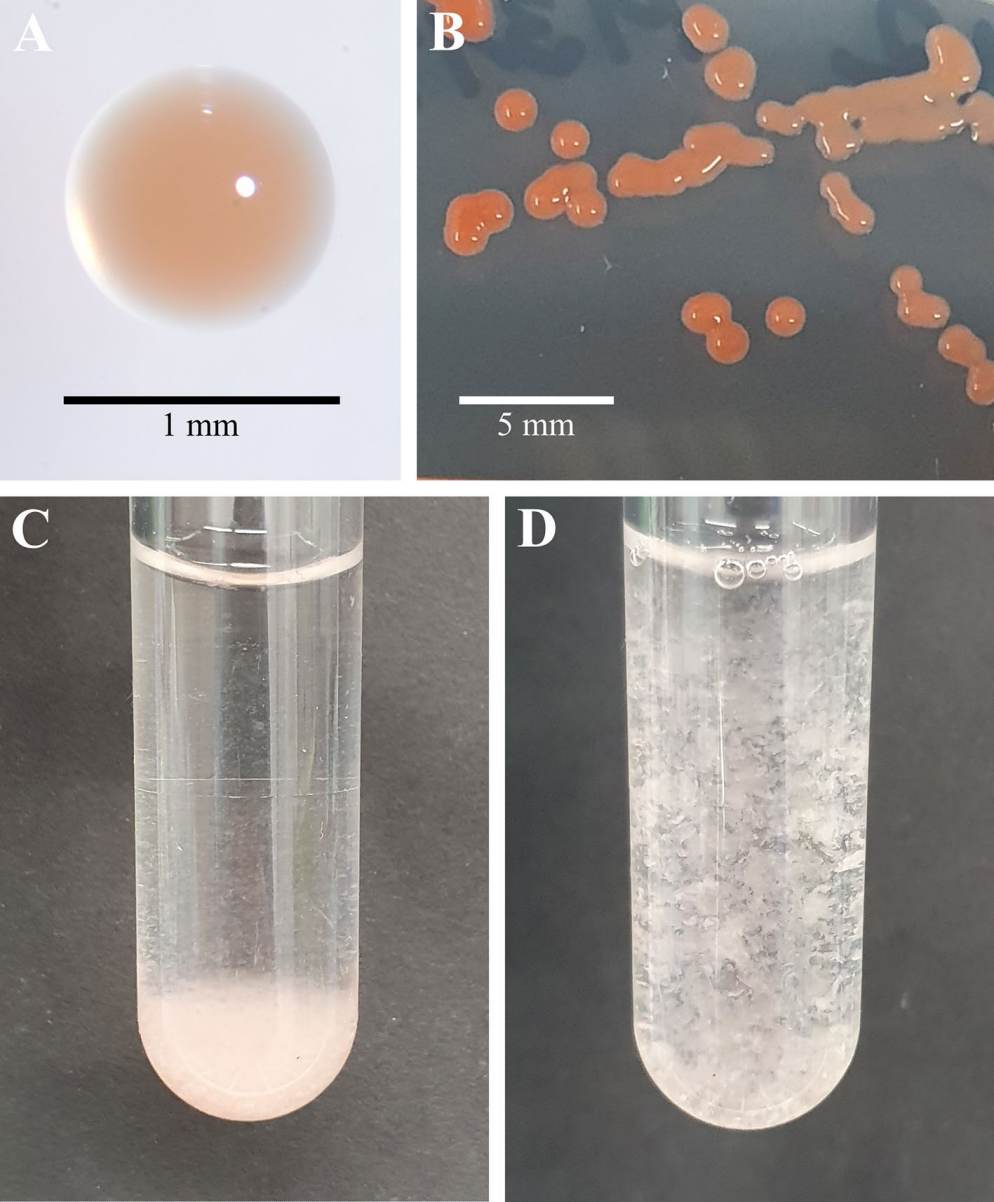
Pigmentation and macroscopic appearance of strain SH412^T^. (**A**) Single colony of the novel isolate incubated at 21 °C on a M31PY plate. The colour shade in the picture represents what is referred to as “salmon-coloured”. (**B**) Colonies of strain SH412^T^ on a M31PY plate, incubated at 21 °C in complete darkness for 20 d and subsequently for three more days next to a window. The colonies display almost the full range of colours that this strain displayed in the current study. However, this picture shows the red/orange shades predominantly. (**C**) Liquid culture grown under constant agitation, the aggregates sedimented at the bottom. (**D**) Aggregates appear like flakes after thoroughly shaking the tube, which also makes OD_600_ measurements impossible.

**Figure 3:**
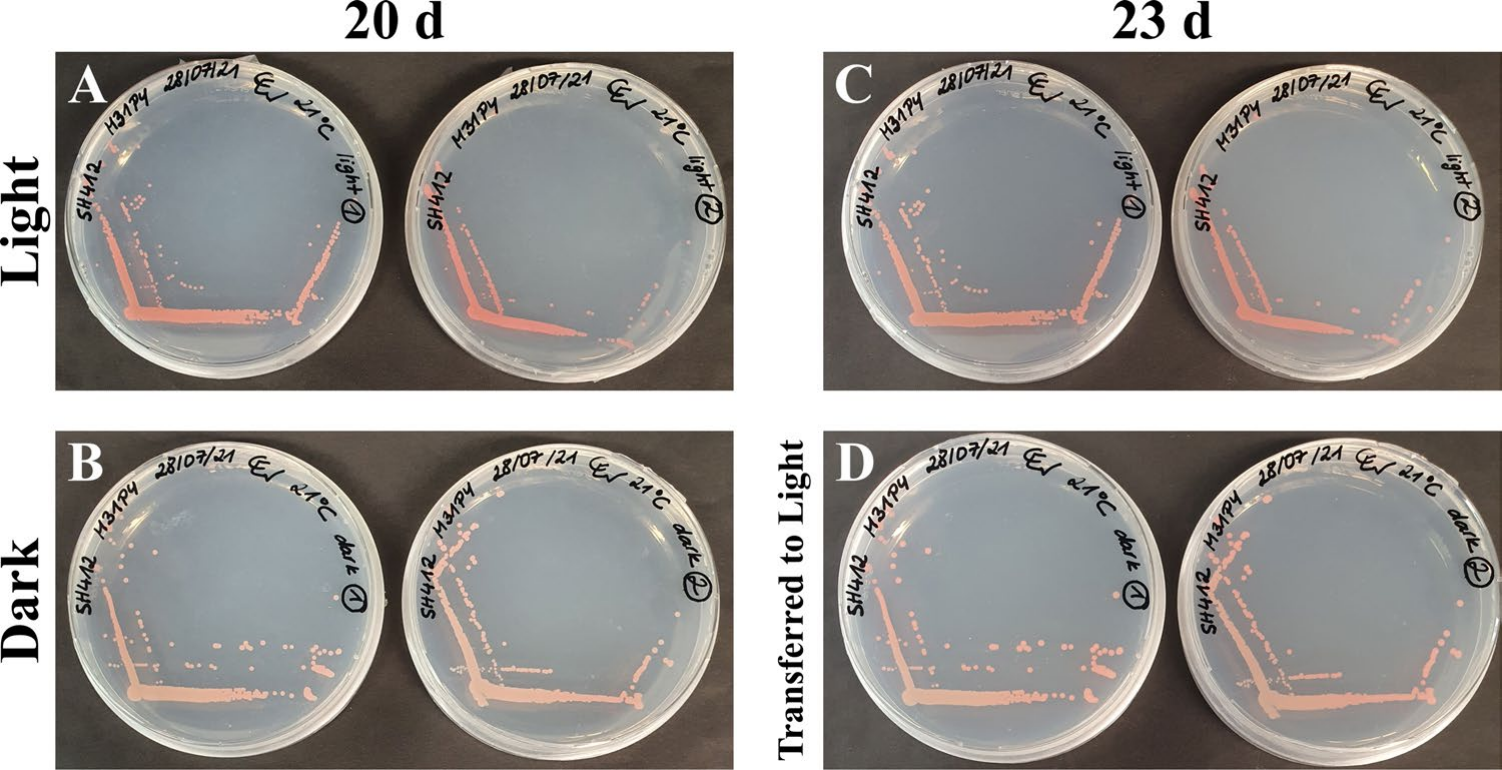
Pigmentation of strain SH412^T^ in dependence on illumination during growth. The plates were incubated for 20 days at 21 °C next to a window (**A**) or in complete darkness (**B**). Notably, the colonies appeared red-pink when grown in light and slightly salmon-coloured upon incubation in darkness. Afterwards, all plates were incubated next to the same window for three more days. Plates that were continuously incubated next to the window did not show any change in colour (**C**). On plates that were previously incubated in darkness, especially single colonies started to turn more red-pink (**D**).

Detailed information on morphology and physiology of strain SH412^T^ is summarised in Table 2. In terms of pigmentation, strain SH412^T^ showed higher similarity with the pink- to red-coloured type strains of the genus *Planctopirus*, while *S. paludicola* MPL7^T^ seems to lack pigment formation. Appearance of strain SH412^T^ varied highly depending on different environmental conditions (Fig. 2), which has not been reported for any of its currently closest relatives. Especially between plates and liquid cultures, the colour of strain SH412^T^ differed significantly. Colonies on plates appeared intensely salmon- to pink- or red-coloured (Fig. 2A,B). Their colour intensity was furthermore found to relate to sun light exposure, with colonies grown in darkness being lighter coloured than colonies grown in daylight (Fig. 3A,B). Light exposure of colonies grown in darkness led to an increase of the pigmentation intensity compared to the control (Fig. 3C,D).

Cells in colonies adhered strongly to each other as well as to the solidified medium, forming round, shiny, and firm colonies (Fig. 2A,B). Colonies transferred from plate to liquid medium lost their colour within a few days and the freshly obtained liquid cultures appeared much lighter in colour. Thereby, most of the cells were part of large, salmon to pale pink, flake-like aggregates, which were impossible to resuspend by agitation (Fig. 2B,C). Aggregates usually emerged in the following manner: Cells attached to the surface of the cultivation tube forming a thin lawn. Especially under agitation, differently sized fragments of this lawn peeled off the walls, thus floating around as aggregates in the medium. Mostly, aggregates started off as thin, mat-like objects, but upon further incubation they disrupted and developed into thicker, more flake-like structures.

**Figure 4:**
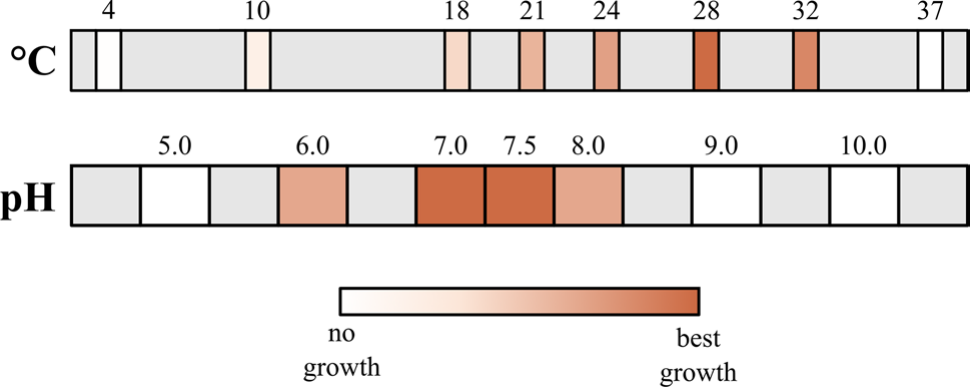
Temperature and pH optimum of strain SH412^T^. The colour code represents the growth of strain SH412^T^ at the different conditions from white (no growth) to red (best growth). However, the evaluation degrees do not represent a linear relationship. Temperature optimum for growth: 28 °C, pH optimum for growth: 7.0–7.5.

**Figure 5:**
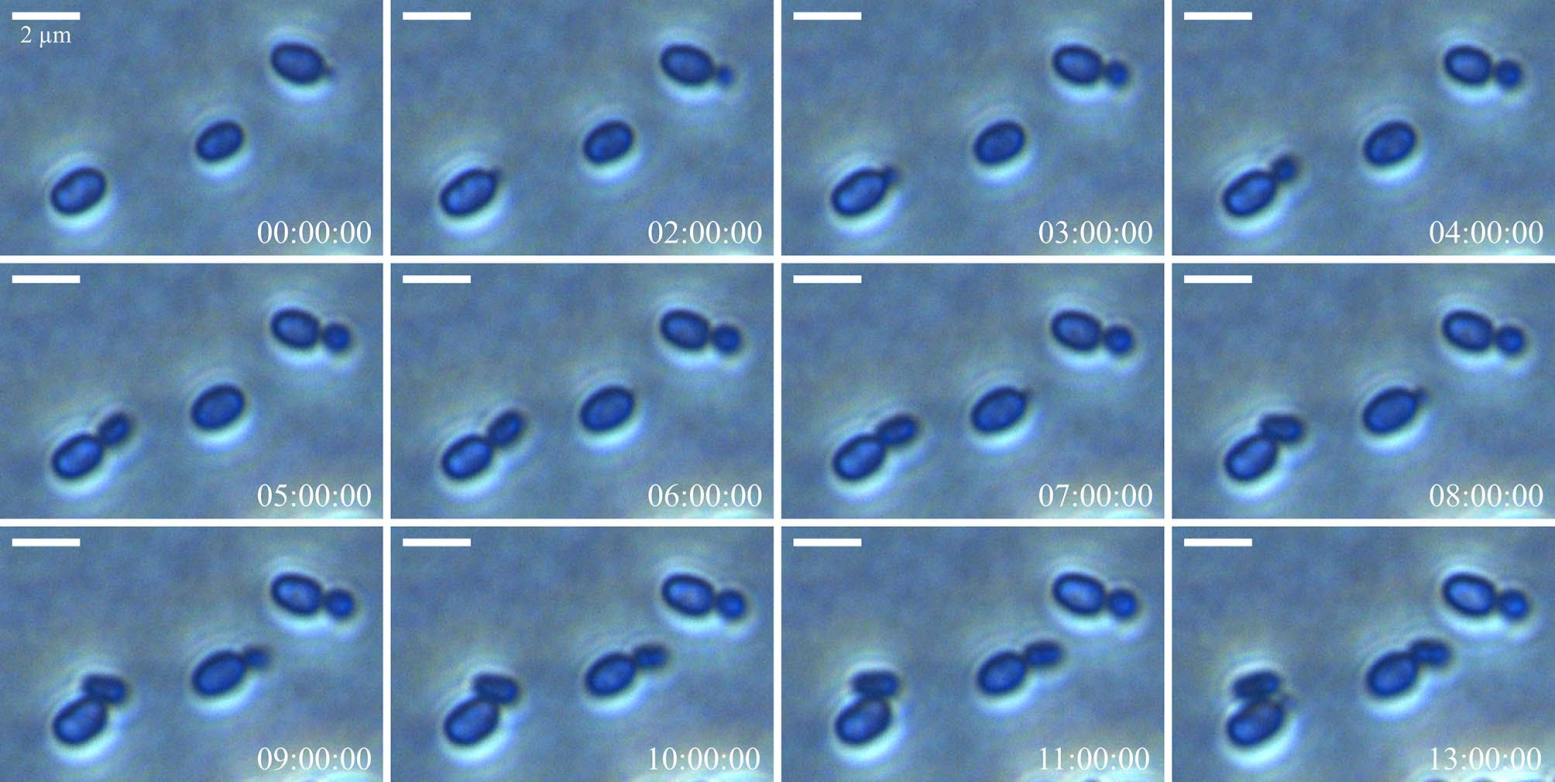
Cell division of strain SH412^T^. Mother cells produce daughter cells by asymmetric cell division (“budding”) at one pole. Phase contrast photos of dividing cells were acquired during time- lapse microscopy in the glass bottom dish setup. Timestamps indicate the hours passed in relation to the first image (left upper corner).

Strain SH412^T^ can grow over a temperature range from 10 to 32 °C and a pH range from 6.0 to 8.0 (Fig. 4). Fastest growth of strain SH412^T^ occurred at 28 °C (evaluated by time until first colonies appeared), however, most biomass was produced at 21 °C (evaluated by colony size). Formation of single colonies took about 10 days at 28 °C and 14 days at 21 °C. After 21 days they had usually reached a diameter of about 2 mm but continued to grow. The pH optimum for growth is between pH 7.0 and 7.5. In that regard, strain SH412^T^ resembles the mesophilic and neutrophilic members of the genus *Planctopirus*, as *S. paludicola* MPL7^T^ is acidophilic. Due to its strong aggregation, growth rate and doubling time could not be determined photometrically. Yet, data from timelapse microscopy analyses suggests a doubling time of around 10 h (Fig. 5).

Strain SH412^T^ turned out to grow under anaerobic conditions almost as well as under aerobic conditions, but colony morphology differed. While the lawn occurred light pink and opaque under aerobic conditions, under anaerobic conditions it was barely coloured and more translucent.

Hence, strain SH412^T^ resembles the type strains of *P. limnophila* DSM 3776^T^ and *S. paludicola* MPL7^T^ regarding its relationship to oxygen, but no morphological differences under anaerobic conditions were reported for these strains. *P. hydrillae* JC280^T^*,* in contrast, is obligate aerobic. Strain SH412^T^ is cytochrome *c* oxidase-positive like the type strains of *P. limnophila* DSM 3776^T^, *S. paludicola* MPL7^T^, and *P. hydrillae* JC280^T^. In contrast to these strains, strain SH412^T^ is catalase-negative (Table 2).

### A new automated pipeline for the determination of the cell size

**Figure 6:**
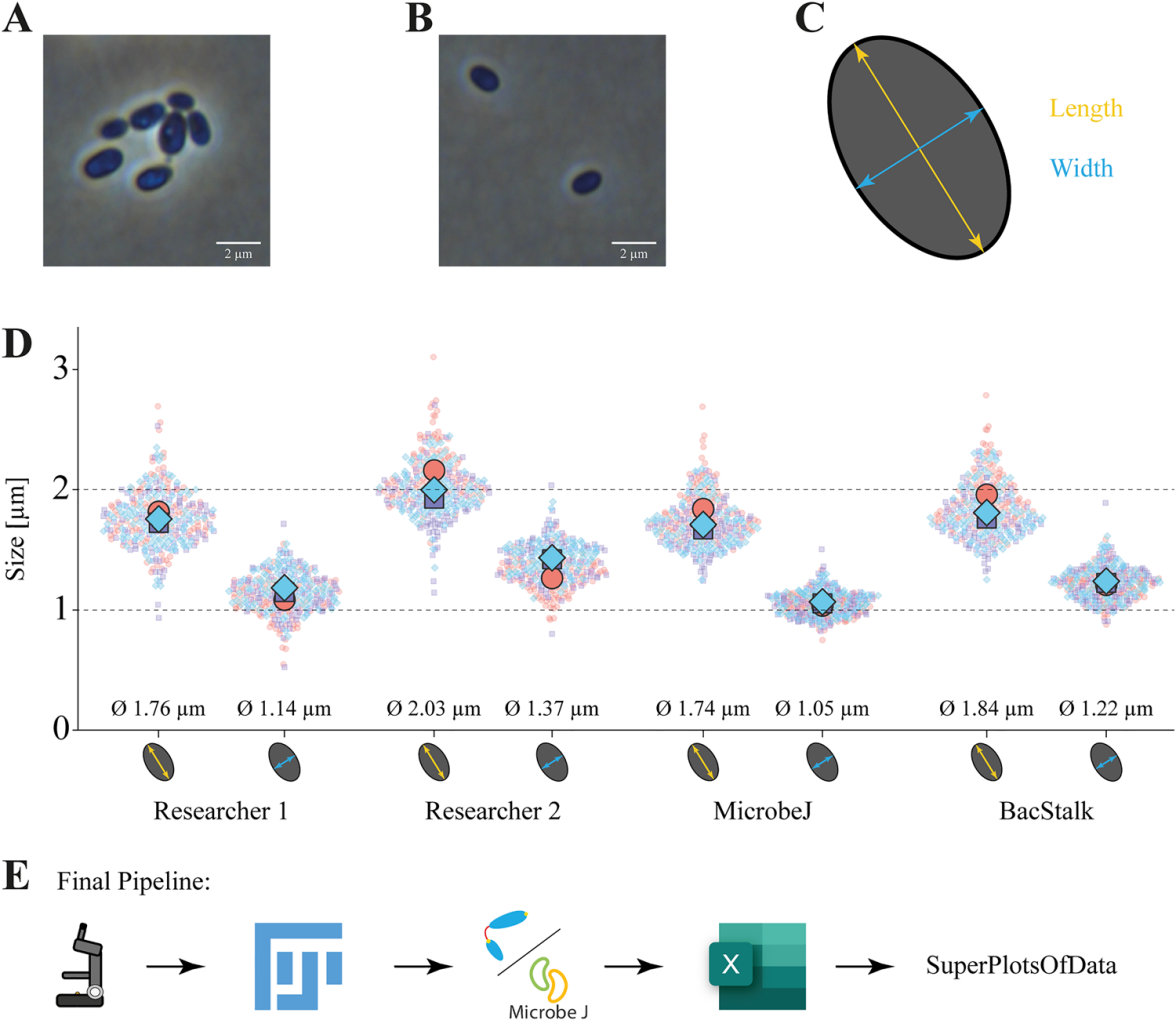
Establishment of a standardised cell size determination pipeline. (**A**) Budding and aggregating cells that were excluded from the analysis due to potential unreliable cell detection by the algorithm. (**B**) Single non-aggregating cells suitable for cell size determination in BacStalk. (**C**) Schematic representation of the obtained cell size parameters. The cell length is defined as the maximal extension of the medial line (yellow) and the cell width equals the maximal distance measured perpendicular to the medial line (blue). (**D**) Comparison between three different cell size determination methods. Two researchers analysed length and width of the exact same 100 cells manually with the line and measurement tools in FIJI, a difference in the mean of up to 0.3 µm in length/width is visible. Each dot represents an individual cell, the three replicates are indicated in blue, purple, and salmon. Mean values are given below the plots. For MicrobeJ and BacStalk only one plot is depicted, as the algorithm is user-independent and thus performs the same when using the same images. Analysis of the exact same cells with different analysis tools yielded comparable mean values with slightly different superplot shapes. (**E**) Final size determination pipeline: obtaining images on the microscope, converting them from native file format to tiff in FIJI, analysing them with BacStalk/MicrobeJ converting the csv files from BacStalk to xlsx files in Excel, and finally plotting the data in SuperPlotsOfData.

During the past few years, we determined the cell size for novel strains of the phylum *Planctomycetota* manually by employing the measurement tool in the Nikon NIS-Elements software^79,80^. However, various semi-automatic applications such as MicrobeJ and BacStalk exist that improve efficiency and reproducibility of the measurement^67,68^. Testing the applicability of BacStalk as well as MicrobeJ for the purpose of describing novel bacterial strains, two independent researchers analysed the very same cells first manually and afterwards employing BacStalk as well as MicrobeJ (Fig. 6). For both measurements taken, researcher 2 obtained slightly higher values than researcher 1 as well as the two softwares. Since both semi-automatic solutions performed equally well and offer the advantage that the algorithms will give the same results when using the same cultivation conditions, software parameters, and input images, we recommend using such semi-automatic programs for cell size determination in strain description articles. Furthermore, high reproducibility can be achieved by imaging bacterial cells of different replicates at a very similar OD_600_ within the mid-exponential phase. For measuring the cell size of strain SH412^T^, we chose BacStalk, as this software, with its fewer options, is easier to employ for untrained researchers. However, for more complex analysis tasks and more complex cell shapes, MicrobeJ appears to be more suitable.

### Microscopic analysis

**Figure 7:**
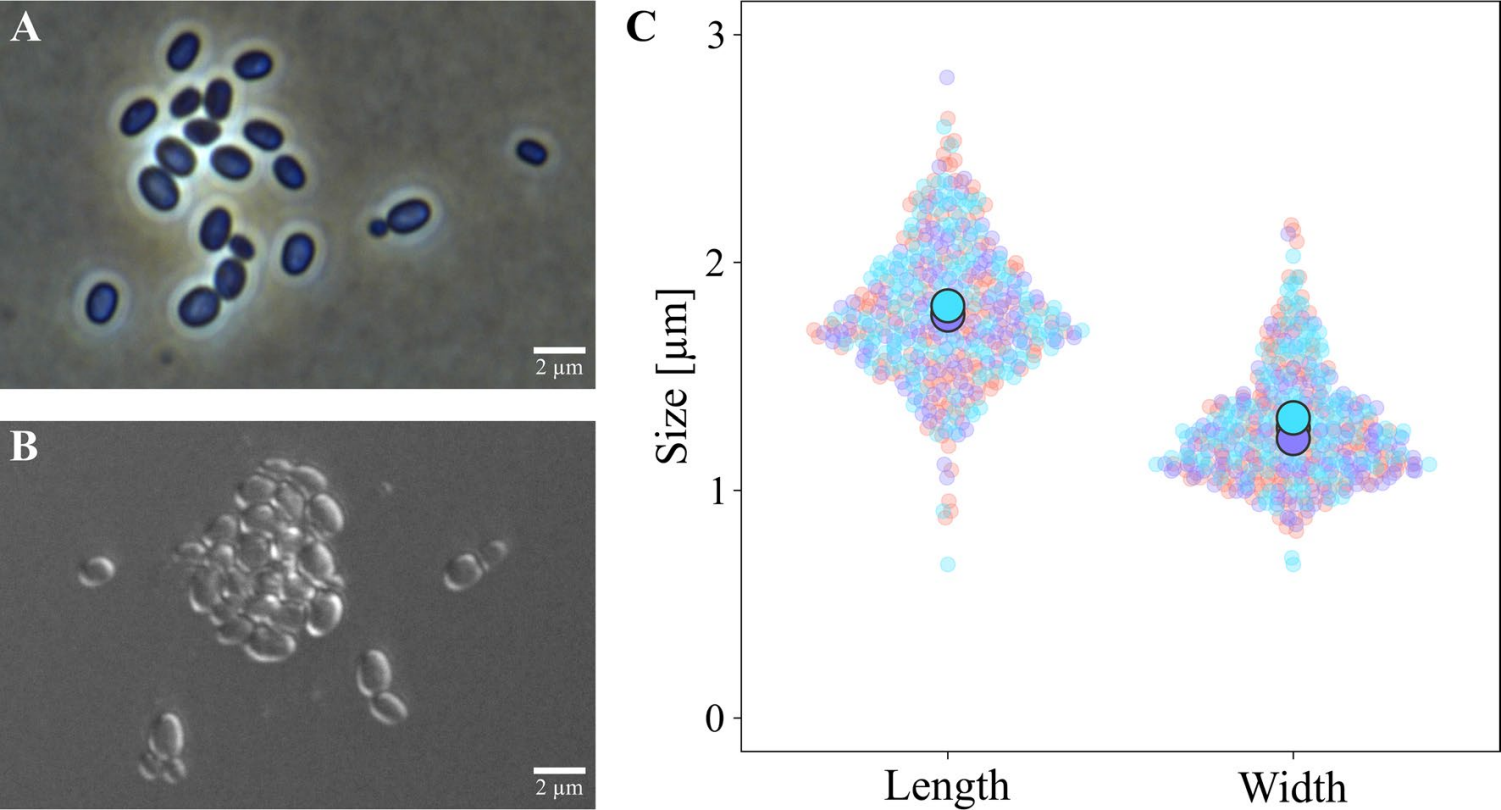
Cellular features of strain SH412^T^. Light microscopy images with (**A**) phase contrast and (**B**) differential interference contrast (DIC) showing single and dividing cells as well as a typical aggregate of strain SH412^T^. (**C**) Superplots of cellular measurements regarding cell length and cell width of strain SH412^T^ for three independent replicates with n = 150, respectively. Each replicate is depicted in a different colour, the replicates’ mean values are indicated by the larger circles overlapping each other.

Cells of strain SH412^T^ measure 0.7–2.2 µm in width × 0.7–2.8 µm in length with most of them being ovoid, but few cells tend to be more spherical (Fig. 7). SH412^T^ divides asymmetrically (polar budding) (Fig. 5 and Fig. 7 A, B) and single (daughter) cells were observed to be motile, while cells in aggregates did not show any movement. Since many genes required for flagellum formation were identified during annotation of the genome using PGAP and all type strains of the currently closest related genera (*Planctopirus, Schlesneria*) were found to possess flagella, it is likely that motility of strain SH412^T^ is conferred by at least one flagellum as well.

In comparison to the four type strains of the genera *Planctopirus* and *Schlesneria*, strain SH412^T^ encompassed a much wider range regarding its cell size. Regarding cell shape, strain SH412^T^ resembles the representatives of the genus *Planctopirus*, which also form ovoid to spherical cells. The variety in cell size is an interesting observation, especially since both length and width can vary with a factor of 2.5 to 3 and thereby spread over an unusually wide range.

These differences found in cell size and motility could be explained by a life-style switch, which has been reported for various planctomycetes, including members of the two currently closest related genera. Producing both swarmer and sessile cells, *P. limnophila* has been shown to possess a life cycle with two distinct cell types^11^. Sessile, stalked mother cells thereby reproduce through budding opposite to the stalked pole and form motile swarmer cells with a single flagellum. The motile daughter cells later attach to surfaces or aggregates, turning into sessile cells that start to divide. Even though a size difference between the two cell types was not explicitly reported for this species, other studies on planctomycetal life cycles reported differences, i.e. daughter cells are about half the size of the mother cells by the time they are released^23,81^. Afterwards, daughter cells increase in size over a defined period before they become sessile. We therefore suggest that such a life cycle is also present in strain SH412^T^.

## Conclusion

Based on both phylogenetic as well as morphological analyses, strain SH412^T^ represents a new species within a new genus belonging to the family *Planctomycetaceae*, for which we propose the name *Planctoellipticum variicoloris* gen. nov., sp. nov.

### Description of *Planctoellipticum* gen. nov.

*Planctoellipticum* (*Planc.to.el.lip’ti.cum*. Gr. masc. adj. *planktos*, wandering, floating; Gr. fem. n. *elleipsis*, ellipse; N.L. neut. n. *Planctoellipticum*, a floating bacterium with an ellipsoid cell shape).

Members of the genus have a Gram-negative cell envelope architecture. Facultatively anaerobic heterotrophs with a mesophilic and neutrophilic growth profile. Cells are ellipsoid and divide asymmetrically. Colonies are pigmented ranging from light pink/salmon to dark red/orange. The DNA G + C content is around 64%. The genus belongs to the family *Planctomycetaceae*, order *Planctomycetales*, class *Planctomycetia*, phylum *Planctomycetota*. The type species of the genus is *Planctoellipticum variicoloris*.

### Description of *Planctoellipticum variicoloris* sp. nov.

*Planctoellipticum variicoloris* (*va.ri.i.co.lo’ris.* L. masc. adj. *varius*, varying; L. masc. n. *color*, colour; N.L. gen. n. *variicoloris*, of varying colour).

Colonies of this species vary in colour from light pink/salmon to dark red/orange in response to sunlight exposure. Cells are ellipsoid/ovoid (length: 0.7–2.8 µm, width 0.7–2.2 µm) and strongly aggregating but not rosette-forming. Crateriform structures, fimbriae and stalks were not observed. Cells are facultatively anaerobic and daughter cells are motile. Cells of the type strain grow at temperatures between 10 and 32 °C (optimum 28 °C) and in a pH range from 6.0 to 8.0 (optimum 7.0–7.5). The type strain is SH412^T^ (= CECT 30430^ T^ = STH00996^T^, the STH number refers to the Jena Microbial Resource Collection JMRC) and was isolated from a wastewater aeration lagoon of a sugar processing plant in Schleswig, Germany. The genome of the type strain has a size of 7.3 Mb and a DNA G + C content of 63.6%.

## Data Availability

The datasets generated and/or analysed during the current study are available from the NCBI GenBank database under the accession numbers OR353748 (16S rRNA gene) and CP130886 (genome).
